# Environmental and Behavioral Drivers of Buruli Ulcer Disease in Selected Communities along the Densu River Basin of Ghana: A Case-Control Study

**DOI:** 10.4269/ajtmh.16-0749

**Published:** 2017-05-03

**Authors:** Samuel Yaw Aboagye, Prince Asare, Isaac Darko Otchere, Eric Koka, George Ekow Mensah, Dzidzo Yirenya-Tawiah, Dorothy Yeboah-Manu

**Affiliations:** 1 Noguchi Memorial Institute for Medical Research, University of Ghana, Accra, Ghana; 2 Institute of Environmental and Sanitation Studies, University of Ghana, Accra, Ghana

## Abstract

The exact route of transmission of *Mycobacterium ulcerans* (MU) (causative agent of Buruli ulcer [BU]), risk factors, and reservoir hosts are not clearly known, although it has been identified as an environmental pathogen. This study assessed potential environmental and behavioral risk factors that influence BU infections. We conducted a case-control study where cases were matched by their demographic characteristics and place of residence. A structured questionnaire was administered to solicit information on the environmental and behavioral factors of participants that may expose them to infection. A total of 176 cases and 176 controls were enrolled into the study. Multivariate conditional logistic regression analysis identified farming in swampy areas (odds ratio [OR] = 4.10, 95% confidence interval [CI] = 3.82–7.18), farming while wearing short clothing (OR = 1,734.1, 95% CI = 68.1–44,120.9), insect bite (OR = 988.3, 95% CI = 31.4–31,115.6), and application of leaves on wounds (OR = 6.23, 95% CI = 4.74–18.11) as potential risk factors. Farming in long clothing (OR = 0.000, 95% CI = 0.00–0.14), washing wound with water and soap (OR = 0.37, 95% CI = 0.29–0.98), and application of adhesive bandage on wounds (OR = 0.31, 95% CI = 0.15–0.82) were found to be protective against BU infection. In the absence of the exact MU transmission mechanisms, education of public in BU-endemic zones on the use of protective clothing during farming activities to limit exposure of the skin and proper wound care management would be essential in the fight against BU.

## Introduction

Buruli ulcer (BU) is a debilitating and necrotizing disease of the skin and soft tissues caused by *Mycobacterium ulcerans* (MU).^1^–^3^ It is the third most important mycobacterial disease globally after tuberculosis and leprosy.^4^ Globally, the disease has been reported in 33 countries, but the greatest disease burden is found mostly in the tropical regions of west and central Africa.^5^,^6^ In west Africa, BU is second after tuberculosis and is the leading mycobacterial disease that affects immunocompetent individuals in highly endemic communities of this region.^1^ The BU disease affects all age groups and sex, but about 50% of cases are found predominantly in children < 15 years of age.^7^–^9^

The first BU case was reported in Ghana in 1971 from a patient in Amasaman, a community along the Densu river basin.^10^ Since then, communities along the river and its tributaries have been extensively surveyed for likelihood of other unidentified BU cases. In other parts of Ghana, such as the Afram valley at Agogo in the Asante Akim North District^11^ and the Amansie West District in Ashanti region,^12^ both along the Offin river basin, a number of BU cases were also reported. The BU disease which was previously perceived to persist only in swampy and tropical rain forest zones in Ghana, however, were found to be in all the 10 administrative regions of Ghana with an overall prevalence of 20.7 per 100,000 of the population,^13^ after a national survey was conducted in 1999.

Though the epidemiology of BU is not fully understood,^9^ studies conducted so far in most endemic regions have linked the occurrence of BU to disturbed environment^8^,^14^,^15^ due to human activities including construction of dams, mining activities, construction of artificial lakes, and extending swamps for growing rice and fish breeding.^8^,^14^–^16^

Since the transmission of BU has remained elusive in regions burdened with BU, identifying potential behavioral and environmental risk factors may contribute to the reduction in the number of BU cases by putting in place required preventive measures. The potential risk factors and protective factors for BU seem to be geographically specific.^17^–^23^ In Cote d'Ivoire for instance, irrigated farming and proximity to remnant rainforest patches have also been associated with higher risk of BU.^24^ In Daloa region of Cote d'Ivoire, Marston and others found participation in farming activities near river bodies as a risk factor for BU infection.^25^ Pouillot and others also identified swamp wading, wearing short, lower-body clothing while farming, living near a cocoa plantation or woods as potential risk factors for BU in Benin.^21^ However, in southeastern Australia, frequent use of insect repellent, wearing of long trousers outdoors, and immediate washing of wounds were protective against BU.^26^ Based on findings from various studies, BU risk factors tend to exhibit regional variations and this could probably be due to differences in geography, environment, and host behavior.

Water, sanitation, and hygiene (WASH) practices form a crucial component in the prevention and control of neglected tropical diseases (NTDs).^27^ The link between some NTDs, including dracunculiasis, trachoma, soil-transmitted helminths, cysticercosis, and schistosomiasis, and WASH interventions has been demonstrated.^28^ However, information on BU and WASH is limited even though BU incidence has been linked to aquatic systems.^14^–^16^ Johnson and others in a recent study evaluated the level of WASH and other associated factors in a BU-endemic district in Benin^29^ and found very low WASH indicators, unimproved water sources for domestic use, and poor sanitation.

In Ghana, studies conducted so far on hygiene practices and other potential risk factors for BU are very limited with variable outcomes.^18^,^30^ Raghunathan and others found wading in river as a risk and bathing with clean water and soap to be protective for BU.^18^ In addition, Kenu and others found the presence of wetland, insect bites in river sites, use of adhesive when injured, and washing in the river as risk factors for BU, and covering of limbs during farming as well as use of alcohol after insect bites as protective factors against BU.^22^ The few case-control studies that have explored BU risk factors were limited by use of only clinical criteria without laboratory confirmation of cases. At the same time, false diagnosis based on clinical criteria alone is known. This study therefore sought to assess the potential environment and behavioral risk factors through a case-control study in selected communities along the Densu river basin of Ghana using only laboratory-confirmed cases.

## Materials and Methods

### Ethics statement.

Ethical clearance for the study was obtained from the institutional review board of the Noguchi Memorial Institute for Medical Research (NMIMR) (Federal-wide Assurance number FWA00001824). Written informed consent was obtained from all individuals that participated in the study. Parents or guardians provided written consent on behalf of all child participants (below 18 years of age).

### Study area.

**Figure 1.**
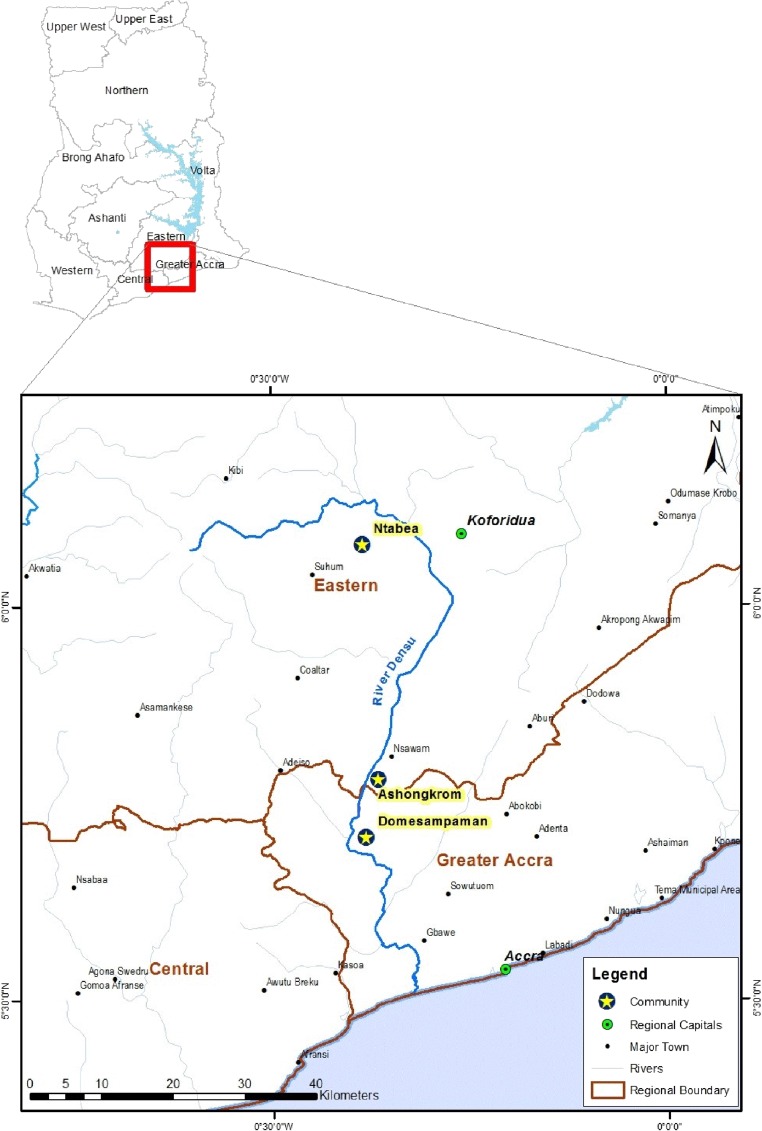
Map of Ghana, showing the Densu river basin and selected communities. Map created with ArcGIS 10.0 using GPS coordinates from the National Buruli Ulcer Control Program.

The study was conducted in three BU-endemic districts, which are East Akim, Akwapim South and Ga West municipality. A common feature of the selected districts is that the Densu river, which runs through all three districts (Figure 1

). The Densu river is 116 km long and is the main source of drinking water supply for the inhabitants. The studied communities are Ntabea in the East Akim District, upstream of the river; Ashongkrom in the Akwapim South District, midstream of the river; and Domesampaman in the Ga West municipality of the Greater Accra Region, downstream of the river. Agriculture is the predominant occupation of the people in the three districts absorbing about 70% of the total population within the districts. These river bodies were selected for the study because extensive disease and seroepidemiological studies have shown high exposure of community members to the MU 18-Kda heat-shock protein 65,^31^–^33^ and unlike other communities in Ghana which depend on passive case report, these sites are active in reporting BU cases to the national BU control program.^13^ Cases and controls were selected from the same communities.

### Case and control definition.

#### Case.

A BU case is defined as any patient presenting with active or inactive BU, diagnosed according to the World Health Organization (WHO) clinical definition,^31^ and confirmed by IS*2404* polymerase chain reaction (PCR) analysis at NMIMR.

#### Control.

A control is defined as an individual who has had no sign or symptoms of active or inactive BU. Controls for children and adult case patients were randomly selected within the communities and matched to cases to the nearest age (±5 years), sex, and residency. Enrollment in the study was voluntary.

### Data collection.

Cases for the study were recruited retrospectively from Noguchi Memorial Institute for Medical Research BU case database. Cases confirmed for BU from January 2013–December 2015 were randomly selected for the study using randomization software (ArcGIS version 10, Esri [Environmental Systems Research Institute], Redlands, CA). Trained resource persons visited homes of the selected cases to seek consent for study participation. Having explained the purpose of the study to the subject and after obtaining consent, a matched control was randomly selected from either the same household as the case subject or neighborhood. After obtaining informed written consent from both cases and controls, we administered standardized questionnaires to participants concerning demography (age, sex, education, and marital status), and environmental (agricultural, fishing, and mining activities) and behavioral practices. We also investigated outdoor behavior and habits including clothing worn, precautions taken against insect bites, how skin traumas were treated, natural fauna with regular contact within households, occupational activities, and activities associated with water bodies. Cases were asked to restrict their responses to the year before the onset of the BU disease or during the past year for control participants. Resource persons fluent in the native language of participants administered the questionnaires with assistance of community health volunteers. All study questionnaires were filled in at the time of the survey and double entries were made during data entry for quality.

### Data analyses.

The responses from the questionnaires were entered in Microsoft Excel 2010 spreadsheet and analyzed using R statistical software (R Development Core Team, 2012, Vienna, Austria). The demography of case and control participants was compared by using two-sample *t* test. Variables that attained significance at a *P* value < 0.05 were retained for multivariate analyses using multiple conditional logistic regressions. We used a step down backward elimination process to identify the factors that are significantly associated with BU and also to control possible confounding factors in the model. *P* values ≤ 0.05 were considered statistically significant.

## Results

### Characteristics of participants.

Overall, equal numbers of cases (176) and controls (176) were matched by age, village, and neighborhood. The demographic characteristics of all BU cases and controls are presented in Table 1. Among the 176 case participants, 86 (49%) were males and 90 (51%) were males. The age of the case participants was in the range 9–51 years, median 28 years, and mean 27 years. The case participants ≤ 15 years of age were 38 (21.6%) and > 15 years were 138 (78.4%) with majority of cases (68; [38%]) belonging to the 25–35 age group. Among the 176 control participants, the age of the controls ranged between 8 and 54 years with a median age of 28 years. Of the 176, 86 (47%) were males and 90 (51%) were females with a mean age of 28 years.

All the cases that participated in the study had been confirmed for BU by PCR following the WHO guidelines and more than half of the cases (115; 65%) presented an ulcerative BU lesion. BU lesions frequently occurred on the lower limbs (77; 44%) followed by the upper limbs (38%). Lesions that occurred on the abdomen were less (8; 4%) compared with the head and trunk (24; 14%). The lesion size presented by case patients were 27 (15%) category I, 55 (31%) category II, and 94 (53%) category III (Table 2).

### Univariate analysis of factors associated with the risk of contracting BU.

#### Socioeconomic factors associated with contracting BU.

Socioeconomic status was assessed by both educational level and occupation type, and these variables were found to be lower in cases than controls. There was a significant association between educational level and risk of BU (odds ratio [OR] = 2.67, 95% confidence interval [CI] = 1.46–5.03). Among the study population, individuals with tertiary level of education (higher education) were protected from developing BU as compared with those without education. Occupation type was also found to influence the chances of contracting BU. Although agricultural farmers were at risk for contracting BU (OR = 2.9, 95% CI = 1.37–3.53), civil servants were protected from BU (OR = 0.24, 95% CI = 0.09–0.56) (Table 3).

### Environmental and behavioral factors for contracting BU.

Cases reported staying in mud walls, having agricultural plantation, and having bushes and woods in their immediate environment more than the controls. The study found that staying in mud walls and mud floors as well as closeness of households to river bodies were negatively associated with BU. Moreover, there was no evidence for association between risk of BU and the presence of agricultural farms, bushes, swamps, and woods in the immediate environment. Also, sources of drinking, bathing, and cooking water were similar for both cases and controls and no evidence was observed for an association between the risk of BU and the water sources.

Washing of clothes around river bodies (OR = 2.12, 95% CI = 1.12–4.11) and passing through the rivers to individual destinations (OR = 2.16, 95% CI = 1.29–3.64) were reported more frequently in cases than the controls and these activities were positively associated with BU (Table 3).

Farming or mining activities in swamps (OR = 3.30, 95% CI = 2.08–5.29) was significantly associated with BU; however, farming around water bodies showed no such association. Although wearing of short upper body clothing (OR = 39.43, 95% CI = 19.91–83.58) and short dress (OR = 14.1, 95% CI = 0.08–25.5) during farming or mining activities were found to be positively associated with BU, the wearing of long upper body cloth (OR = 0.31, 95% CI = 0.14–0.63), and trousers (OR = 0.28, 95% CI = 0.17–0.45) during such activities were found to be protective for BU (Table 3).

Wrapping wounds with leaves were reported more frequently in case patients than controls and it was positively associated with BU (OR = 3.91, 95% CI = 2.32–6.74). Washing of wounds with water and soap (OR = 0.52, 95% CI = 0.31–0.85) and applying adhesive bandage (OR = 0.42, 95% CI = 0.26–0.66) on wounds were found to be protective for BU.

### Insect bites.

The risk of contracting BU was significantly associated with insect bites (OR = 227.58, 95% CI = 58.02–1,999.04) and use of insect net at night appeared to be protective for BU (OR = 0.25 95% CI = 0.14–0.42) (Table 3).

### Multivariate analysis of factors associated with the risk of contracting BU.

The multivariate analyses (conditional logistic regressions) found none of the socioeconomic factors as a potential risk for contracting BU. However, some environmental and behavioral factors were retained. Farming activities in swamps (OR = 4.10, 95% CI = 3.82–7.18), wearing of short upper body clothes (OR = 1,734.1, 95% CI = 68.1–44,120.9), wearing of short lower body clothes (OR = 14.4, 95% CI = 1.25–165.7), insect bite (OR = 988.3, 95% CI = 31.4–31,115.6), and application of leaves on wounds (OR = 6.23, 95% CI = 4.74–18.11) were found to be risk factors for contracting BU. Moreover, farming in long upper body cloth (OR = 0.000, 95% CI = 0.00–0.14), farming in trousers (OR = 0.58, 95% CI = 0.21–0.97), washing wounds with water and soap (OR = 0.37, 95% CI = 0.29–0.98), and application of adhesive bandage on wounds (OR = 0.31, 95% CI = 0.15–0.82) were found to be protective against BU disease (Table 4).

## Discussion

The exact route of MU transmission is unclear, although it is commonly presumed that infection takes place through physical contact with environmental reservoirs. This study aimed to identify potential environmental and behavioral risk factors for BU disease in some selected BU-endemic communities along the Densu river basin. We found that 1) farming in swampy areas, farming in short upper and lower body clothes, and wrapping of wounds with leaves increased the risk of contracting the BU disease and 2) farming in long upper body clothes, wearing trousers, washing wounds with water and soap, and application of adhesive bandages to wounds decreased the risk of contracting the BU disease.

The major findings from our study are in agreement with several other epidemiological studies that have been conducted in BU-burdened regions to identify risk factors associated with BU disease.^20^,^21^,^23^,^26^,^34^–^36^ Subsistence agriculture (44%) is the major socioeconomic activity of the BU cases in the study areas. Moreover, most cases reported farming more frequently in swampy areas than controls. Our findings using both univariate and conditional logistic regression analyses show that farming in swampy areas is a major risk factor for BU. Agricultural activities, such as farming particularly in swampy areas, puts cases in constant contact with moist/watery soil. Tian and others recently showed that MU strains can survive in soil for 4 months suggesting that BU could be acquired through contact of open wounds with watery soil as a transient source of infection.^37^ Moreover, Aboagye and others recently isolated MU from soil in a BU-endemic community along the Densu river basin of Ghana.^38^ Bratschi and others also indicated that MU may persist for many months in decaying organic matter under water.^39^ These findings clearly confirm that farming in swampy areas is a potential risk factor for BU. Furthermore, we found wearing of long clothing (upper and lower body) during farming activities to be protective against BU, whereas wearing of short clothing (upper and lower body) was a risk factor for contracting BU. This finding is consistent with many other studies conducted in both Ghana and Cote d'Ivoire.^18^,^22^,^25^ The hot weather conditions that are frequently experienced in most African countries encourage farmers to wear less protective clothes during farming to work efficiently. This behavior results in long periods of skin exposure which is likely to facilitate infection, which is evident by different studies that majority of BU lesions are located on lower limbs than on abdomen.^13^,^35^,^39^–^41^ In addition, exposed BU lesions could also serve as a source of infection as has been hypothesized that humans with large active ulcerative BU lesions could shed off the bacteria into the environment in the course of their daily activities.^39^

Likewise, we also found washing of wounds with water and soap and application of adhesive bandages to offer some protection against BU, which is consistent with findings by previous studies.^18^,^23^ These protective mechanisms could be due to the washing off of the contaminating pathogens with water and soap of the surface of the skin, whereas the bandages serve as cover for the open wounds thereby limiting entry of pathogens.

One of the proposed theories is that MU may be transmitted through an insect bite.^42^ Studies conducted in both Ghana and Benin by Portaels and others detected MU DNA in water bugs belonging to the families Naucoridae and Belostomatidae.^42^ Moreover, Marsollier and others isolated MU from wild aquatic insects collected from a zone in the Daloa region of Ivory Coast and also showed that MU may be transmitted to laboratory mice by the bite of aquatic bugs (Naucoridae) that are infected with this organism.^43^ In southeastern Australia, Quek and others, in a case-control study, reported that more cases than control individuals recalled that they were bitten by mosquitoes on the lower extremities.^26^ The authors provided evidence that implicated mosquitoes in the transmission of MU in southeastern Australia.^26^ Johnson and others also screened mosquitoes in a small town during a BU outbreak in southeastern Australia and confirmed the presence of MU in a subset of pools by detection of three PCR targets (IS*2404*, IS*2606*, and Ketoreductase)^44^ In the present study, we found insect bite to be a potential risk for BU as confirmed by both the univariate and conditional logistic regression analyses which supports the proposed MU transmission hypothesis, although no study has confirmed PCR targets from mosquitoes in Ghana as has been shown in southeastern Australia.

In contrast to findings by Pouillot and others,^21^ we found the application of leaves on open wounds to be a potential risk factor for contracting BU. Stinear and others identified aquatic plants as a possible reservoir of this pathogen.^45^ More recently, Aboagye and others also detected MU DNA from vegetation biofilm from BU-endemic community along the Densu river basin of Ghana.^38^ Aquatic plants, such as algae for instance, are able to secrete many organic compounds, such as amino acids and polysaccharides, which are in turn used by bacteria as substrates for growth.^46^–^49^ Genotype analysis by Marsollier and others showed that plant-associated MU had the same profile as isolates recovered in the same region from aquatic insects and clinical specimens, an observation that seems to implicate aquatic plants as possible reservoir of MU.^50^ Direct application of herbal preparations containing MU to wounds could then be a source of infection, increasing the risk of suffering from BU.

The preventive behaviors outlined in this study, particularly washing with soap and clean water are already a major component of the WASH program.^51^ This has been integrated into the school systems in most deprived rural communities of Ghana through a partnership between Ghana Health Service, Ghana Education service, and UNICEF toward meeting the millennium development goals, goal 7C.^51^ The partnership has made it possible for provision of boreholes and other sanitary facilities to improve hygiene conditions within the schools and the communities. Moreover, some nongovernmental organizations also provide sanitation supplies to a number of selected rural deprived schools in an effort to enhance hygiene practices. The introduction of the WASH concept facilitated in the eradication of guinea worm in northern Ghana as well as other endemic areas of Africa.^52^–^54^ It is therefore feasible for this approach to be used toward the fight against BU disease in endemic areas. Furthermore, even though Ghana sits on the equator with almost year-round warm weather conditions, it is not far-fetched to recommend the wearing of long clothing to prevent direct contact of possible contaminated surfaces.

As part of our extension services aimed at increasing access to health care for BU patients and support for the health-care system (clinics) in the management of BU, we developed a number of interventions including community outreach to enhance early case detection and provision of transportation for BU patients,^55^ which helped to improve BU management in the Obom treatment center. However, since these projects are temporal, we strongly recommend local governments and the health systems to take up these intervention measures and make provision for boreholes in rural communities that lack clean and safe water. We are of the view that sustenance of these interventions would contribute immensely to the fight against the BU disease.

This study, like other case-control studies is not exempted from limitations. Cases most often live with the BU disease for long periods before seeking medical assistance; therefore, there is the potential for recall bias that may influence case responses thereby impacting negatively on the study.

In conclusion, our study identified potential risk factors for BU along the Densu river basin. We found that farming in short clothes (upper and lower body) in swampy areas and wrapping of wounds with leaves were major risk factors of contracting the BU, whereas wearing of long clothes (upper and lower body) during farming, washing of wounds with water and soap, and application of adhesive bandages to wounds protective for BU. In the absence of the exact MU transmission mechanisms, education of public on the use of protective clothing during farming activities and proper wound care management are essential for the fight against BU.

## Figures and Tables

**Table 1:** Demographic characteristics of cases and controls

Characteristics	No. of cases *n* (%)	No. of controls *n* (%)
	176 (100)	176 (100)
Sex		
Male	86 (49)	86 (49)
Female	90 (51)	90 (51)
Age in years (median, range)	28 (9–51)	28 (8–54)
≤ 15	38 (22)	34 (19)
16–24	33 (19)	34 (19)
25–35	68 (38)	77 (44)
≥36	37 (21)	31 (18)
Marital status		
Single	99 (56)	100 (57)
Married	76 (43)	74 (42)
Divorced	1 (0.5)	2 (1)

**Table 2:** Clinical characteristics of BU cases

Characteristics	No. of cases *n* (%)
	176 (100)
Lesion type	
Ulcerative	115 (65)
Nonulcerative	61 (35)
Location of lesion	
Lower limbs	77 (44)
Upper limbs	67 (38)
Head and trunk	24 (14)
Abdomen	8 (4)
Category of lesion	
Category I (< 5 cm)	27 (15)
Category II (5–15 cm)	55 (31)
Category III (> 15 cm)	94 (53)

**Table 3:** Univariate analysis of selected variables for Buruli ulcer disease in communities along the Densu river basin, Community-matched case-control study

Parameters	No. of cases (*N*, %)	No. of controls (*N*, %)	Univariate OR (95% CI)	*P* value
Socioeconomic status				
Primary	90 (51)	86 (49)	1.09 (0.71–1.70)	0.7492
Secondary	34 (19)	48 (27)	073 (0.43–1.25)	0.2525
Tertiary	7 (4)	25 (14)	0.25 (0.09–0.62)	< 0.0014
No education	45 (26)	20 (11)	2.67 (1.46–5.03)	< 0.0009
Occupation				
Artisan	3 (2)	8 (5)	1.09	0.7492
Student	44 (25)	54 (31)	0.75	0.2845
Farmer	77 (44)	46 (26)	2.19 (1.37–3.53)	< 0.001
Trader	14 (8)	18 (10)	0.76	0.5787
Fisherman/woman	5 (3)	3 (2)	1.68	0.7234
Miner	25 (14)	18 (10)	1.45	0.3288
Civil servant	8 (5)	29 (16)	0.24 (0.09–0.56)	< 0.001
Household environment				
Mud wall: yes/no	77 (44)	68 (39)		0.448
Mud floor: yes/no	38 (22)	39 (22)		0.699
Within < 1 km vs. > 1 km to river basin	46 (26)	42 (24)		0.924
Agricultural plantation in the immediate environment: yes/no	66 (38)	65 (37)		0.912
Bush in the immediate environment: yes/no	158 (90)	152 (86)		0.324
Swamp in the immediate environment: yes/no	88 (50)	95 (54)		0.595
Woods in the immediate environment: yes/no	79 (45)	69 (39)		0.280
Source of drinking water				
Borehole	140 (80)	133 (76)		0.369
River/stream	14 (8)	10 (6)		0.526
Sachet	21 (11)	32 (18)		0.097
Hand dug well	1 (0.6)	1 (0.6)		1.000
Source of water for bathing				
Borehole	154 (88)	154 (88)		0.893
River/stream	19 (10)	20 (11)		1.000
Hand dug well	3 (2)	2 (1)		1.000
Source of water for cooking				
Borehole	153 (87)	154 (88)		0.986
River/stream	21 (12)	20 (11)		1.000
Hand dug well	2 (1)	2 (1)		1.000
Domestic activities				
Washing clothes around site: yes/no	36 (20)	19 (10)	2.12 (1.12–4.11)	0.013
Passing through river to destination: yes/no	60 (34)	34 (19)	2.16 (1.29–3.64)	0.004
Agricultural/mining activities				
Farming/mining in swamps: yes/no	126 (71)	76 (43)	3.30 (2.08–5.29)	< 0.001
Farming/ mining around river/stream: yes/no	46 (26)	35 (20)		0.164
Wearing long upper body cloth to farm/mine: yes/no	13 (7)	36 (20)	0.31 (0.14–0.63)	< 0.001
Wearing short upper body cloth to farm/mine: yes/no	163 (93)	42 (24)	39.43 (19.91–83.58)	< 0.001
Wearing trouser to farm/mine: yes/no	80 (45)	132 (75)	0.28 (0.17–0.45)	< 0.001
Wearing short dress to farm/mine: yes/no	80 (45)	150 (85)	14.1 (0.08–25.5)	< 0.001
Insect bite				
Receive insect bites in river/home: yes/no	174 (99)	48 (27)	227.58 (58.02–1,999.04)	< 0.001
Use of mosquito net: yes/no	28 (16)	132 (75)	0.25 (0.14–0.42)	< 0.001
Use of mosquito coil: yes/no	82 (46)	94 (53)		0.286
Wound management				
Washing wounds with water/soap: yes/no	37 (21)	60 (34)	0.52 (0.31–0.85)	0.012
Robbing wounds with alcohol: yes/no	23 (13)	38 (22)	0.55 (0.29–0.99)	0.067
Wrapping wounds with leaves: yes/no	75 (43)	28 (16)	3.91 (2.32–6.74)	< 0.001
Applying adhesive bandage on wounds: yes/no	56 (32)	93 (53)	0.42 (0.26–0.66)	< 0.001

**Table 4:** Multivariate analysis of selected variables for Buruli ulcer disease in communities along the Densu river basin, Community-matched case-control study

Parameters	Multivariate OR (95% CI)	*P* value
Farming in swamps	4.10 (3.82–7.18)	0.001
Farming in long upper body cloth	0.000 (0.00–0.14)	0.032
Farming in short upper body cloth	1,734.1 (68.1–44,120.9)	< 0.001
Farming in trousers	0.001 (0.000–0.14)	< 0.001
Farming in short lower body cloth	14.4 (1.25–165.7)	< 0.000
Insect bites in rivers or home	988.3 (31.4–31,115.6)	0.001
Washing wounds with water and soap	0.37 (0.29–0.98)	0.001
Wrapping wounds with leaves	6.23 (4.74–18.11)	< 0.001
Applying adhesive bandage on wounds	0.31 (0.15–0.82)	0.036
